# Multiple Biatrial Thromboses in a Patient with End-Stage Nonobstructive Hypertrophic Cardiomyopathy

**Published:** 2016-07-06

**Authors:** Ali Hosseinsabet

**Affiliations:** Tehran Heart Center, Tehran University of Medical Sciences, Tehran, Iran.

**Keywords:** *Thrombosis*, *Cardiomyopathy, hypertrophic*, *Atrial fibrillation*

A 72-year-old patient with a history of nonobstructive hypertrophic cardiomyopathy type III and atrial fibrillation of several years’ duration presented to the emergency department with dyspnea at rest (New York Heart Association functional class IV). The patient had been using metoprolol, digoxin, furosemide, and warfarin and had a history of significant variations in the International Normalized Ratio (INR). Physical examinations revealed irregular pulse (80 bpm), pulmonary fine rales in the half lower part of both lungs, and lower extremity edema (2+). The patient was admitted for the treatment of decompensated heart failure. Transthoracic echocardiography revealed normal left ventricular end-diastolic diameter and volume and reduced left ventricular systolic function (ejection fraction = 47%, by the modified Simpsons method) with maximal wall thickness in the mid anteroseptal portion (26 mm) and a large mass in the left atrium. Next, transesophageal echocardiography was done and it showed 1 fixed large thrombosis (50 × 40 mm) attached to the roof of the left atrium and another thrombosis with an echo-lucent center attached to the roof of the right atrium. Additionally, the left atrial appendage was obliterated by clot (Figure 1 and Figure 2). There was a significant smoky pattern in both atriums. The patient was discharged after the symptoms were controlled and the therapeutic INR level (3 - 3.5) was achieved and was recommended to have close follow-up on the INR. The presence of concomitant left and right atrial thromboses in patients with hypertrophic cardiomyopathy is unusual, but it should be kept in mind.

**Figure 1:**
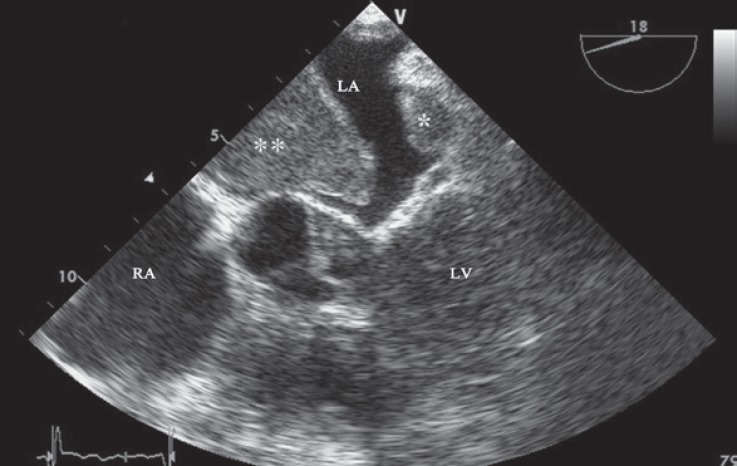
Left atrial appendage thrombosis (*) and left atrial thrombosis (**) are illustrated in transesophageal echocardiography (upper esophageal view).

LA, Left atrium; LV, Left ventricle; RA, Right atrium

**Figure 2:**
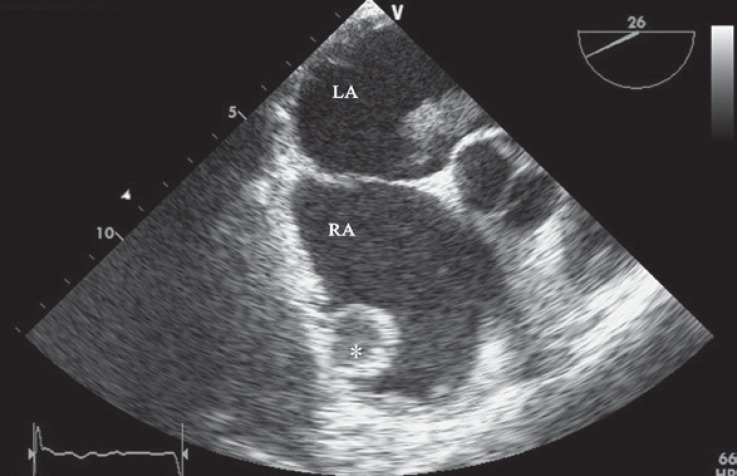
Right atrial thrombosis (*) is depicted in transesophageal echocardiography (upper esophageal view).

LA, Left atrium; RA, Right atrium

***To watch the following videos, please refer to the relevant URLs:***

Video 1. Left atrial thrombosis and left atrial appendage thrombosis

http://jthc.tums.ac.ir/index.php/jthc/article/view/401/467

Video 2. Right atrial thrombosis

http://jthc.tums.ac.ir/index.php/jthc/article/view/401/468

## Notes:

***This paper should be cited as: ***Hosseinsabet A. Multiple Biatrial Thromboses in a Patient with End-Stage Nonobstructive Hypertrophic Cardiomyopathy. J Teh Univ Heart Ctr 2015;11(3):157-158.

